# Long-Term Prognosis and Impact Factors of Metoprolol Treatment in Children with Vasovagal Syncope

**DOI:** 10.3390/biomedicines14010075

**Published:** 2025-12-30

**Authors:** Jing Wang, Ping Liu, Yuli Wang, Junbao Du, Ying Liao, Hongfang Jin

**Affiliations:** 1 Department of Pediatrics, Peking University First Hospital, Beijing 100034, China; 2 State Key Laboratory of Vascular Homeostasis and Remodeling, Peking University, Beijing 100191, China

**Keywords:** vasovagal syncope, metoprolol, children, heart rate variability, long-term prognosis

## Abstract

**Objective:** To investigate long-term prognosis and impact factors in children with vasovagal syncope (VVS) receiving metoprolol therapy. **Methods:** This retrospective study included children with VVS who underwent metoprolol therapy at the Pediatric Syncope Unit of Peking University First Hospital between January 2012 and November 2023. Baseline demographic data, pre-treatment indices, including head-up tilt test (HUTT) and 24 h Holter monitoring, were collected. All participants received standardized metoprolol therapy for a minimum duration of one month. Follow-up was conducted between June and July 2025, with syncope recurrence as the primary endpoint. Multivariable Cox proportional hazards regression analysis was performed to identify independent impact factors of prognosis and to construct a Prognostic Risk Score (PRS) model. The model’s performance was rigorously validated through receiver operating characteristic (ROC) curve analysis, decision curve analysis (DCA), and Bootstrap resampling (1000 iterations). Furthermore, children were stratified into high- and low-risk groups based on median PRS values. Kaplan–Meier survival analysis was then performed to assess the model’s discriminative efficacy. **Results:** This study included 97 children diagnosed with VVS. The median duration of metoprolol therapy was 2.5 months (interquartile range [IQR]: 2.0–3.0 months), with a median follow-up period of 59 months (IQR: 25.5–72 months). During follow-up, syncope recurrence was observed in 37 patients, while 60 patients remained symptom-free. COX regression analysis showed that time-domain indices of heart rate variability (HRV), including the standard deviation of all NN intervals (SDNN) and the triangular index (TR), as well as the frequency-domain index of HRV very low frequency (VLF), were relative factors of the long-term prognosis in children with VVS treated with metoprolol. Based on the above three identified factors, the PRS model was calculated as: PRS = 0.03 × SDNN − 0.02 × VLF − 0.1 × TR. ROC showed that the area under the curve (AUC) for discriminative power related to long-term prognosis was 0.808 (*p* < 0.01). The cumulative recurrence rate of symptoms in the high-risk score group was significantly higher than that in the low-risk score group (*p* < 0.01). The DCA curve demonstrated the clinical applicability of the model. Bootstrap internal verification indicated high stability, with the bias-corrected and accelerated (Bca) confidence interval (CI) of the C index ranging from 0.71 to 0.89. **Conclusions:** After metoprolol treatment, 38.1% of children with VVS experienced syncope recurrence during a median follow-up period of 59 months. Baseline HRV index, SDNN, TR, and VLF were identified as factors associated with the long-term prognosis of children with VVS treated with metoprolol. The PRS model based on the above indices demonstrated good value in linking to the individual long-term prognosis.

## 1. Introduction

Syncope is a common emergency in childhood, affecting up to 15–25% of children at least once in their lives. The primary causes of syncope in children are neurally mediated syncope (NMS) and cardiogenic syncope (CS), with vasovagal syncope (VVS) being the predominant form of NMS, accounting for 60–80% of pediatric syncope cases [1,2]. Children with VVS may experience abrupt bradycardia and/or a drop in blood pressure when standing for prolonged periods, especially in hot environments, or when exposed to emotional stress. This leads to insufficient cerebral perfusion and subsequent fainting. The unpredictability and suddenness of syncope significantly increase the risk of trauma in children, while recurrent episodes pose a substantial threat to their physical and mental health [3,4]. Currently, the main treatment methods of VVS include non-pharmacological therapy and pharmacotherapy. Non-pharmacological therapy is the primary approach, and pharmacotherapy is reserved for children who do not respond well to non-pharmacological interventions [5,6]. Emerging evidence suggests that some children with VVS exhibit baseline autonomic dysfunction, characterized by heightened sympathetic activity, which may further deteriorate during orthostatic challenges, especially prior to syncopal events [7,8,9]. Therefore, metoprolol, a β-adrenergic receptor blocker, has the potential to inhibit sympathetic nerve activation and is used to treat children with VVS.

However, the reported efficacy of metoprolol in pediatric VVS remains inconsistent, underscoring the urgent need to identify clinical profiles that indicate responsiveness. Most previous work has focused on short-term outcomes, evaluating surrogate markers of sympathetic activation, such as 24 h urine norepinephrine level, left ventricular ejection fraction, baroreflex sensitivity, and the heart rate increment during HUTT, over treatment periods of 2–4 months [10,11,12,13]. Whether these indices, or any others, inform long-term prognosis after metoprolol treatment remains unknown.

HRV is widely accepted as a non-invasive window on the balance between the sympathetic and vagus nerves. Both time- and frequency-domain indices are routinely employed to quantify autonomic tone. In children with VVS, however, the literature presents conflicting portraits. Several groups have reported reductions in time-domain HRV indices adjacent NN intervals > 50 ms (pNN50), root mean square of the successful NN interval differences (rMSSD), and frequency-domain HRV component high frequency (HF), together with an increase in low frequency (LF), indicating heightened sympathetic tone and diminished vagal activity [14]. Others observed lower SDNN during the first 5 min of HUTT, again interpreted as evidence of sympathetic predominance [15,16]. Conversely, Shim S et al. found that, at rest, children with VVS exhibit higher SDNN, rMSSD, and HF power than those in controls, implying a baseline parasympathetic excess [17]. Collectively, these data confirm that VVS encompasses heterogeneous autonomic phenotypes and underscore the potential of HRV parameters to stratify this heterogeneity for therapeutic decision-making [18,19].

Therefore, this study was designed to collect baseline HRV-related indicators in children with VVS prior to metoprolol treatment and to explore their association with long-term prognosis, aiming to identify the prognostic factors in this metoprolol-treated cohort.

## 2. Materials and Methods

### 2.1. Study Population

This was a single-center, retrospective cohort study conducted in the Pediatric Syncope Unit of Peking University First Hospital. This study was open-label (non-blinded). Children were eligible for inclusion if they met the following criteria:

VVS was diagnosed when all of the following criteria were met [11]: (1) clear triggers (e.g., prolonged standing, sudden postural change from prone/squat to upright position, emotional stress, or crowded, poorly ventilated environments); (2) a documented history of syncope episodes; (3) a positive HUTT; and (4) exclusion of other causes of transient loss of consciousness [1].

Inclusion criteria: the patients were eligible if they (1) were admitted to the Pediatric Syncope Unit of Peking University First Hospital between January 2012 and November 2023 who first diagnosed as VVS; (2) were aged 4–18 years; (3) were treated with health-education plus metoprolol; and (4) had complete baseline data, including demographics, full medical history, physical examination and comprehensive 24 h Holter monitoring.

Exclusion criteria: the patients were excluded if they (1) had transient loss of consciousness of unknown or non-VVS etiology (e.g., cardiac syncope, epilepsy, or psychogenic pseudosyncope); (2) had other conditions that influence HRV, such as thyroid dysfunction, anemia, diabetes, or acute infection; (3) received any alternative treatments during follow-up (e.g., oral rehydration salts, upright training, midodrine hydrochloride, pyridostigmine, fludrocortisone); (4) demonstrated poor compliance or a treatment duration less than one month; or (5) had undergone prior non-pharmacological or pharmacological treatment for VVS before admission [12].

This study was approved by the Ethics Committee of Peking University First Hospital (approval number: 2025R0063-0002). For all participants under 8 years of age, their guardians provided verbal consent via telephone; for those between 8 and 18 years old, both the participants themselves and their guardians completed the informed consent process by phone.

We collected the following baseline variables: demographic characteristics [age, sex, and body mass index (BMI)]; hemodynamic parameters (supine systolic blood pressure, supine diastolic blood pressure, and resting heart rate); and HRV-related indices. HRV indices included both time-domain and frequency-domain measures. Time-domain indices comprised: SDNN, standard deviation of the averages of NN intervals in all 5 min segments of the entire recording (SDANN), mean of the standard deviation of NN intervals for each 5 min segment (ASDNN), rMSSD, pNN50, and TR. Frequency-domain indicators included: LF, HF, and VLF.

### 2.2. Head-Up Tilt Test

Before HUTT, any medication with autonomic effects was withheld for at least 5 half-lives, and participants fasted at least 4 h. After 10 min of quiet supine rest in a dimly lit, temperature-controlled room, baseline blood pressure, heart rate, and electrocardiogram (ECG) were recorded. Subjects were then tilted to 60° on an electronic tilt table (SHUT-100A, STANDARD, Jiangyin, China) and continuously monitored with a multi-parameter monitor (Dash 2000, GE Healthcare, New York, NY, USA) and beat-to-beat blood pressure monitor (Finometer PRO, FMS, Amsterdam, The Netherlands). The test was terminated when a positive response occurred or after the full 45 min protocol [20].

A positive HUTT response was defined as the occurrence of syncope or presyncope accompanied by at least one of the following: (1) blood pressure drop: systolic blood pressure ≤ 80 mmHg, diastolic blood pressure ≤ 50 mmHg or mean blood pressure decrease ≥ 25%; (2) heart rate drop: <75 beats per minute (bpm) in children aged 4–6 years, <65 bpm in children aged 6–8 years, or <60 bpm in children over 8 years old; (3) sinus arrest or junctional escape rhythm; and (4) transient second-degree or higher atrioventricular block, or asystole ≥ 3 s. VVS hemodynamic subtypes were classified as follows: (1) vasodepressor: significant decrease in blood pressure without concomitant bradycardia; (2) cardioinhibitory: significant bradycardia without concomitant hypotension; and (3) mixed: simultaneous significant falls in both heart rate and blood pressure [1,21].

### 2.3. Analysis of Heart Rate Variability Indicators

HRV analysis was assessed from continuous recording from 3-channel or 12-channel Holter monitors (CT-083S, Beneware, Hangzhou, China; H-Scribe7.0, Mortara, Milwaukee, WI, USA). Participants were instructed to pursue normal daily activities while avoiding prolonged strenuous exercise, and any drugs known to influence autonomic tone were withheld for at least 48 h beforehand. ECG data were exported via Mortara H-Scribe 7.0 software (Mortara Instruments) and analyzed with the BES-ECG Pro Dynamic Electrocardiogram Analysis System [22].

Time-domain HRV indices comprised SDNN, SDANN, ASDNN, rMSSD, pNN50, and TR. Frequency-domain components of HRV were derived by Fast Fourier Transform method and included LF (0.04–0.15 Hz), HF (0.15–0.4 Hz), and VLF (0.003–0.04 Hz).

### 2.4. Treatment Regimen and the Protocol of Follow-Up

All children diagnosed with VVS received oral metoprolol at an initial dose of 0.5–1.0 mg/kg per day, given in two divided doses, with a maximum daily dose not exceeding 50 mg. The treatment duration was 1–3 months, and doses were titrated according to body weight [1].

From June to July 2025, all enrolled patients were followed up via telephone by designated physicians, with a median follow-up time of 59 months (IQR: 25.5–72 months). The primary focus included the occurrence of syncope symptoms during the follow-up period, with the endpoint event defined as the first recurrence of syncope during follow-up. All follow-up information for the pediatric patients was recorded using an Excel (Microsoft^®^ Office Home & Student 2021, Microsoft Corporation, Redmond, WA, USA) spreadsheet, explicitly documenting the patient’s medical record number, follow-up date, syncope recurrence status (yes/no), time to first recurrence (accurate to the month), triggers of recurrence, accompanying symptoms, and medication compliance. The time to first recurrence was used as the basis for survival analysis. For patients without recurrence, the last follow-up time was recorded and included in the analysis as censored data. All time measurements were recorded in “months”.

### 2.5. Establishment of PRS Model

Least Absolute Shrinkage and Selection Operator (LASSO) regression was the main process to finalize the variable selection. To avoid the computational load and model overfitting, before LASSO regression, we first performed a preliminary variable screening using univariate Cox regression analysis. Variables with potential statistical associations with syncope recurrence (*p* < 0.50) were selected and then incorporated into the subsequent LASSO regression for further analysis. Variables meeting this criterion were forwarded to a LASSO regression model with ten-fold cross-validation to select the optimal regularization parameter (lambda [λ] min), defined as the value yielding the minimum cross-validated error. This procedure maximized model generalizability and minimized overfitting. The final multivariable Cox model (significance, two-sided *p* < 0.05) retained the variables identified by LASSO and was used to build the PRS model. Hazard ratios (HR) with 95% confidence intervals (CI) were estimated for each retained factor, and individual PRS values were calculated by summing the products of each selected variable and its corresponding Cox regression coefficient (β).

### 2.6. Evaluation and Validation of PRS Model

The PRS model’s efficacy was evaluated with ROC analysis; the AUC quantified its ability to distinguish children who did or did not experience syncope recurrence, with values approaching 1.0 indicating superior discriminatory power. DCA was performed to assess the model’s clinical net benefit across a spectrum of threshold probabilities, explicitly weighing the trade-offs between intervention risks and benefits. Internal validation was carried out with 1000 bootstrap resamples to obtain bias-corrected 95% CI for all performance metrics.

### 2.7. Stratification Based on the PRS Model

Using the median PRS value as the cut-off, the cohort was divided into high-risk and low-risk groups. Kaplan-Meier curves with log-rank testing demonstrated a marked separation in cumulative syncope recurrence rates between strata, visually confirming the model’s ability to discriminate long-term prognosis.

### 2.8. Statistical Analysis

Data were analyzed with SPSS 27.0 (IBM, Armonk, NY, USA) and R (version 4.2.3). Normality of continuous variables was assessed using the Shapiro–Wilk test. Normally distributed data are expressed as mean ± standard deviation; non-normally distributed data are presented as median with interquartile range (IQR).

R statistical analyses were performed using the following packages: survival (Cox proportional hazards regression models), glmnet (LASSO regression), survminer (Kaplan–Meier plots), rmda (DCA), pROC (ROC and AUC), bootstrap (bootstrap internal validation), ggplot2 (data visualization), and mice (multiple imputation). In this study, missing TR values were imputed by multiple regression interpolation. All analyses were performed on both the complete case dataset and the imputed dataset. Parameter estimates differed by less than 5%, confirming that the results were not materially influenced by the missing-data strategy.

## 3. Results

### 3.1. Baseline Characteristics of the Study Population

We retrospectively enrolled 113 children diagnosed with VVS who attended the Pediatric Syncope Unit of Peking University First Hospital between January 2012 and November 2023. After a median follow-up of 59 months (IQR 25.5–72). Sixteen patients were lost to follow-up (attrition rate: 14%), leaving 97 children (48 boys, 49 girls) for final analysis. Syncope recurred in 37 patients (38.1%), while 60 (61.9%) remained symptom-free throughout follow-up.

Subtype distribution was vasodepressor in 86.6% (84/97), cardioinhibitory in 7.2% (7/97), and mixed in 6.2% (6/97). An identifiable trigger, most commonly postural changes, emotional stress, or crowded environments, was reported in 82.5% (80/97). Notably, prodromal symptoms preceded 92.8% (90/97) of initial episodes. The most frequent prodromal manifestations were visual disturbances (blackouts, blurred vision), vestibular symptoms (dizziness, tinnitus), and autonomic signs (pallor, weakness), often occurring in combination. Before treatment, the median number of syncopal episodes was 4 (IQR 2–6). Additional baseline characteristics are summarized in Table 1.

### 3.2. Factors Affecting Long-Term Prognosis of Children with Vasovagal Syncope Treated with Metoprolol and Modeling of Prognostic Risk Score

**Figure 1:**
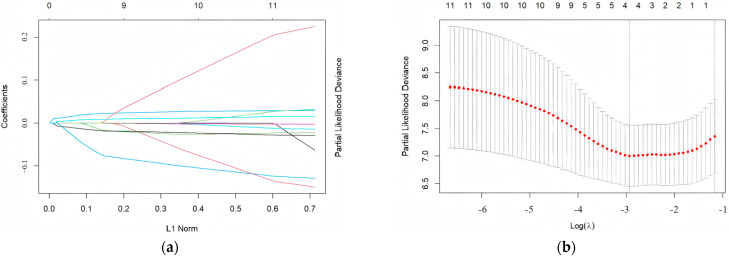
LASSO regression for screening prognostic factors in pediatric vasovagal syncope treated with metoprolol. (**a**) LASSO coefficient trajectory plot: This panel illustrates the coefficient trajectories of independent variables, including HRV metrics, across varying regularization strengths (λ). The *x*-axis represents the L1 norm (sum of absolute coefficients), corresponding to increasing regularization intensity, while the *y*-axis displays standardized regression coefficients. Progressive regularization compresses non-essential variable coefficients to zero, achieving feature selection and model parsimony. The colored lines represent different variables. (**b**) LASSO regression cross-validation plot: The line formed by the red dots in the center represents the mean of the partial likelihood deviance under cross-validation. The left dashed vertical line denotes the λ value yielding the minimum MSE (λ.min), whereas the right line indicates the largest λ within one standard error of the minimum MSE (λ.1se). Partial likelihood deviance: A measure of model fit. In this study, λ.min was selected to retain four HRV-related variables (SDNN, ASDNN, TR, VLF) for subsequent multivariable Cox proportional hazards regression. This strategy preserved critical HRV parameters while eliminating non-specific variables, thereby optimizing model generalizability without compromising performance. ASDNN: Average standard deviation of 5 min NN intervals; HRV: heart rate variability; MSE: mean squared error; SDNN: standard deviation of all NN intervals; VLF: very low frequency power.

The primary endpoint was syncope recurrence during follow-up in children with VVS who received metoprolol. Among the 97 evaluable patients, 37 children experienced recurrent syncope. Seventeen candidate variables were first screened by univariate Cox proportional hazards regression analysis (Table 2). Eleven variables with a *p* < 0.5, systolic blood pressure, heart rate, height, SDNN, rMSSD, ASDNN, pNN50, TR, VLF, and LF/HF, were advanced to a LASSO regression model with ten-fold cross-validation. The optimal penalty parameter (λ.min) retained four variables: SDNN, ASDNN, VLF, and TR (Figure 1). These four variables were then entered into a multivariable Cox model (Table 3). Three emerged as independent prognostic factors: baseline SDNN (3% increased recurrence risk per 1 ms increment; HR 1.03, 95% CI 1.01–1.06), VLF (2% risk reduction per 1 ms increment; HR 0.98, 95% CI 0.95–0.99), and TR (9% risk reduction per 1-unit increase; HR 0.91, 95% CI 0.85–0.97). Using the corresponding regression coefficients, we constructed a PRS: PRS = 0.03 × SDNN − 0.02 × VLF − 0.1 × TR.

### 3.3. Evaluation and Validation of Prognostic Risk Score Model

**Figure 2:**
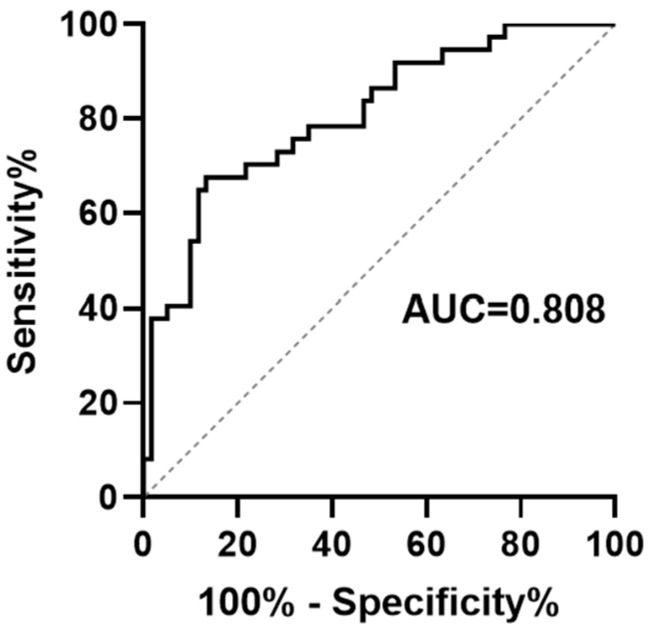
ROC evaluating performance of the PRS model for indicating long-term prognosis in pediatric vasovagal syncope treated with metoprolol. The ROC curve illustrates the efficiency of the PRS model. The *x*-axis represents 1-specificity, and the *y*-axis denotes sensitivity for indicating long-term outcomes following metoprolol treatment in pediatric VVS. The 45-degree reference line indicates equal sensitivity and specificity. The black ROC curve corresponds to the PRS model, demonstrating an AUC of 0.808. AUC: area under the curve; PRS: prognostic risk score; ROC: receiver operating characteristic; VVS: vasovagal syncope.

**Figure 3:**
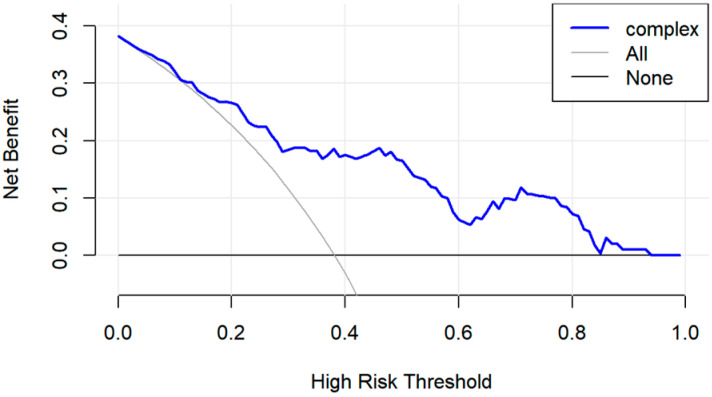
DCA of the PRS model for indicating long-term prognosis in pediatric vasovagal syncope treated with metoprolol. This DCA evaluates the clinical utility of the PRS model. The *y*-axis represents the net benefit derived from therapeutic interventions, while the *x*-axis indicates the high-risk threshold probability. The blue curve (“PRS-guided metoprolol therapy”) reflects the net benefit of treatment decisions based on the PRS model, the gray curve (“Treat all”) denotes the net benefit of administering metoprolol to all patients, and the black horizontal line (“Treat none”) corresponds to the net benefit of withholding treatment universally. When the threshold probability ranges from 0.1 to 1.0, the PRS-guided strategy demonstrates a net benefit range of 0.01–0.4, outperforming both alternative approaches. DCA: decision curve analysis; PRS: prognostic risk score.

To quantify the PRS model’s discriminative power related to long-term outcomes in pediatric VVS patients treated with metoprolol, we subjected it to ROC analysis. The AUC reached 0.808 (Figure 2), indicating a good discrimination between children who will and will not experience recurrent syncope on metoprolol. DCA curve was then used to assess clinical utility. Across threshold probabilities from 0.1 to 1.0, the PRS-guided strategy generated a higher net benefit than either the “treat-all” or “treat-none” approaches (Figure 3). This implies that basing therapeutic and monitoring decisions on PRS yields meaningful clinical advantage over current empirical strategies.

Internal validation was performed with bootstrap resampling (1000 iterations). The BCa 95% CI for the C-index was 0.71–0.89, confirming both statistical significance (C-index > 0.5) and stability of the model estimates. These findings indicate that the PRS model reliably discriminates between high- and low-risk pediatric VVS patients, supporting its potential utility for individualized prognosis assessment in clinical practice.

### 3.4. Stratified Analysis of Long-Term Prognosis of Children with Vasovagal Syncope Treated with Metoprolol Based on Prognostic Risk Score Modeling

**Figure 4:**
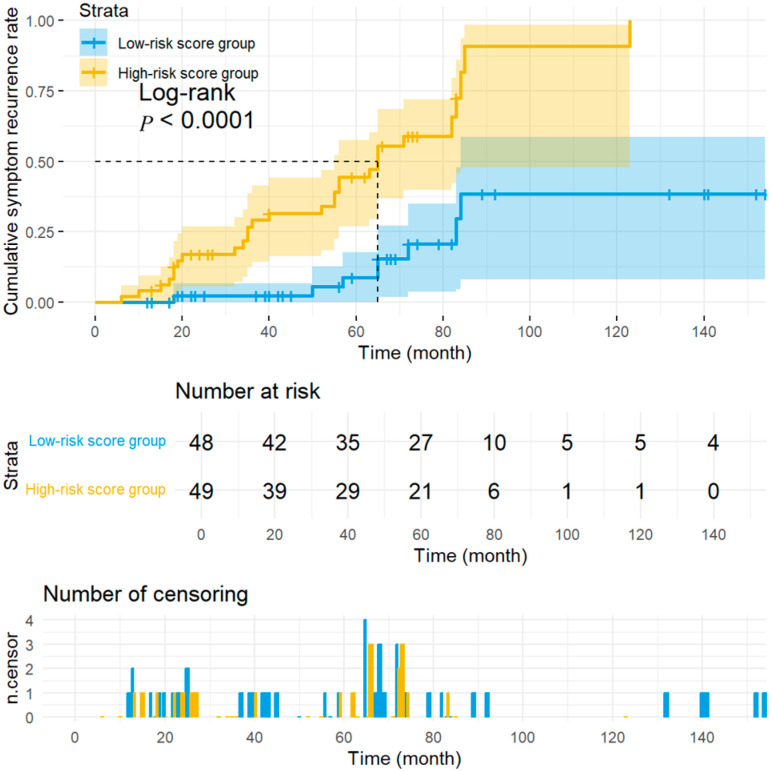
Kaplan–Meier curves for long-term prognosis assessment of metoprolol-treated pediatric vasovagal syncope stratified by risk score groups. The Kaplan–Meier curves compare cumulative symptom recurrence rates between high-risk and low-risk score groups. The *x*-axis represents follow-up time (months), and the *y*-axis indicates the cumulative symptom recurrence rate. The yellow curve corresponds to the high-risk score group, while the blue curve denotes the low-risk score group. A significant divergence in recurrence rates was observed between the two groups (log-rank test: *p* < 0.0001). The dashed line intersection point in the plot indicates the time point (in the abscissa), at which the cumulative symptom recurrence rate of children in both groups approaches 50% (in the ordinate). Risk table: Displays the number of patients at risk (remaining in follow-up) within each subgroup at specified time points. Censoring events: Represent cases where symptom recurrence did not occur before data censoring. The censoring distribution across time points is provided to evaluate data completeness. The number of censorings is shown at the bottom, in which blue bars correspond to the low-risk score group, while yellow bars represent the high-risk score group; the height of the bars at different time points corresponds to the number of censored cases in the respective risk group at that time point.

To validate the discriminatory capacity of the PRS model, scores were computed for all 97 children according to the formula: PRS =0.03 × SDNN − 0.02 × VLF − 0.1 × TR. Using the median PRS of 0.36 as the cut-off, patients were assigned to a high-risk group (*n* = 49) or a low-risk group (*n* = 48) based on the median PRS threshold (0.36). Kaplan–Meier analysis with log-rank testing revealed a significant separation in cumulative syncope recurrence between strata (log-rank test: *p* < 0.01), confirming that the PRS robustly identifies children at increased long-term risk (Figure 4).

## 4. Discussion

In this study, we found that the baseline HRV indices, including the time-domain HRV index SDNN and TR, and frequency-domain component VLF, were independently associated with syncope recurrence in children with VVS who received metoprolol during long-term follow-up. A PRS model built from these three parameters stratified patients into high- and low-risk groups for recurrent syncope. The AUC of the ROC for model assessment was 0.808, indicating good discriminative power. Kaplan–Meier curves revealed significant differences in cumulative symptom recurrence rate between the high- and low-risk groups stratified by the PRS model, with clear separation of the curves. BCa internal validation further demonstrated that the 95% CI for the C-index was 0.71–0.89, without significant attenuation. DCA curve indicated favorable net clinical benefit across a broad range of threshold probabilities, further supporting the model’s discriminative power, referring to the long-term prognosis in children with VVS treated by metoprolol.

Autonomic dysfunction is central to the pathophysiology of VVS. Under the triggers such as prolonged standing, compensatory sympathetic in some VVS children generates excessive myocardial contractility. This hypercontractile state stimulates ventricular mechanoreceptors, triggering a paradoxical vagal surge. The resulting bradycardia or transient asystole, often coupled with peripheral vasodilation, precipitates cerebral hypoperfusion and syncope. Metoprolol, a beta-blocker, has been commonly used for patients with VVS for a long period. Its therapeutic mechanism is based on the observation that, during an episode, some patients with VVS experience excessive sympathetic activation prior to the consequence of vagal excitement, leading to the sudden drop in heart rate and/or blood pressure. This activation is supposed to be an important precursor that triggers excitation of central vagal nuclei. Therefore, in such patients, theoretically, metoprolol may reduce the risk of syncopal episodes by selectively blocking myocardial β-receptor-mediated catecholaminergic signaling, thereby suppressing cardiac overexcitability [20,23]. Previous studies suggested that β-blockers demonstrated significant efficacy in the treatment of adult VVS, including higher rates of conversion to a negative HUTT result [24,25], lower syncope recurrence rates [26], and superior efficacy with a favorable side effect profile even at low doses [27,28]. Similarly, metoprolol was also shown to be effective in treating VVS [29,30], with one study in children reporting a cure rate of 60.61% [31]. However, one study noted that 29% of pediatric patients experienced at least one recurrent syncopal episode after metoprolol treatment [32], suggesting variable effectiveness. Furthermore, studies have shown that β-blockers primarily attenuated syncopal episodes in pediatric VVS by blunting sympathetic-mediated activation of cardiac mechanoreceptors. Nevertheless, published efficacy rates for pediatric VVS are approximately 60%, and several trials have failed to demonstrate a significant reduction in recurrence [33,34]. In a cohort followed for 48 ± 28 months, Kouakam et al. documented a 32% relapse rate despite the β-blocker therapy [35]. This may be related to the complex pathogenesis of VVS, meaning that not all VVS patients exhibit a state of sympathetic overexcitation. Additionally, potential side effects associated with metoprolol require vigilance, such as bradycardia and heart block, hypotension, bronchospasm, and masking of hypoglycemia symptoms. Hence, strict attention must be paid to its indications and contraindications when using metoprolol for VVS treatment. In our study, after a median of 59 (IQR 25.5–72) months of metoprolol treatment, syncope recurred in 38.1% of VVS children.

These divergent outcomes likely resulted from the multifactorial pathogenesis of pediatric VVS. In addition to autonomic dysregulation, relative hypovolemia and impaired peripheral vasoconstrictive responses can act independently or synergistically to precipitate syncope. Therefore, reliable biomarkers that related to therapeutic outcomes following metoprolol treatment are urgently needed to guide individualized treatment decisions [36].

The time-domain HRV parameter SDNN is widely accepted as an assessment of the balance between sympathetic and vagal activity. Previous studies suggest that SDNN serves as an indicator of sympathetic and vagal regulation, with greater SDNN values representing suppressed sympathetic tone and vagal dominance [17]. According to the PRS model in the present cohort study, every 1 ms increment in baseline SDNN was associated with a 3% rise in the hazard of syncope relapse in children with VVS after metoprolol treatment. Conversely, children with lower pretreatment SDNN experienced significantly fewer recurrences over long-term follow-up. These data may support the hypothesis that patients with higher baseline sympathetic tone can derive more benefit from β-blockers.

TR is believed to be a fundamental parameter reflecting the overall HRV, calculated as the ratio of the total number of NN intervals to the height of the NN-interval histogram. Previous studies have shown that TR, as a predictor of cardiovascular mortality in patients with atrial fibrillation, reflects the balance between sympathetic and vagal function [37]. Schwartz et al. demonstrated that a reduced TR reflects autonomic imbalance [38]. Hämmerle et al. established that TR < 14.29 independently predicted both cardiovascular death and all-cause mortality (HR, 1.70; 95% CI, 1.12–2.59; *p* = 0.01) [39]. We found that baseline TR was associated with syncope recurrence in children with VVS after metoprolol treatment. Specifically, each 1-unit increment in TR was associated with a 9% reduction in the hazard of syncope recurrence. This indicated that children with a higher pretreatment TR seemed to experience a more favorable long-term prognosis. This benefit may reflect a better-preserved autonomic balance, though the precise mechanisms remain to be elucidated.

Frequency-domain HRV indicators include VLF, LF, and HF. Several studies indicate that VLF is a sensitive marker of autonomic status, with VVS children exhibiting higher VLF than healthy controls [40]. Study demonstrated that VLF was significantly higher in the HUTT-positive group compared with the HUTT-negative pediatric patients, suggesting elevated sympathetic tone in the former group. Studies also demonstrated that VLF, a component of HRV, is primarily regulated by the sympathetic nervous system [41,42]. Collectively, an increase in VLF may suggest relatively heightened sympathetic excitability [43]. In the present study, each 1-ms increment in VLF corresponded to an approximately 2% reduction in the hazard of syncope recurrence, indicating that children with higher pretreatment VLF may have a better long-term prognosis, consistent with the previous studies.

Previous studies have developed models for predicting the short-term efficacy of metoprolol in treating children with VVS [44,45]. For instance, Cui et al. established a scoring model with the formula Logit(P) = −3.053 + 0.099 × ΔHR + 0.069 × QTcd − 0.034 × SDNN (ΔHR, increased heart rate during positive response in HUTT; QTcd, corrected QT dispersion). Using a cutoff value of 2.5 points, this model predicted the efficacy of metoprolol in children with VVS over 1–3 months with a sensitivity of 93.6%, specificity of 80.9%, and accuracy of 87.7% [44]. Another scoring model developed by Du et al., formulated as Logit(p) = −14.511 − 0.267 × age + 1.725 × MPV (MPV, mean platelet volume), used a cutoff value of 0.5 points and demonstrated a sensitivity of 88.1% and specificity of 73.3% in predicting the efficacy of metoprolol in pediatric VVS patients during a 1 month to 3 months therapy [45].

In addition, some studies have focused on the relatively long-term outcomes and associated risk factors of metoprolol treatment in children with VVS. Zhang et al. followed 28 children with VVS who received either metoprolol or conventional treatment for one year over a mean period of 22 ± 10 months. They used Kaplan–Meier curve analysis to assess the time to first syncope recurrence and compared groups using the log-rank test. The results indicate that the recurrence of VVS in children and adolescents treated with metoprolol was similar to that in patients receiving conventional treatment without discussing the factors associated with the outcome [34]. Research by Tao et al. showed that after a median treatment period of 9.0 months (IQR: 4.8, 19.1) with metoprolol, 29% of children patients with VVS experienced one or more syncopal episodes during follow-up. Pretreatment frequency of syncope was identified as an independent risk factor for recurrence in these patients (hazard ratio = 1.027, 95% confidence interval 1.009–1.045, *p* = 0.003) [32]. In a 54-month follow-up study of 170 children with orthostatic intolerance treated with oral rehydration salts combined with metoprolol (including 48 VVS patients), Li et al. found that pretreatment symptom score was a risk factor affecting treatment efficacy [46]. These risk factors predicting recurrence mainly involved medical history without relatively objective physiological indexes that can indicate the therapeutic mechanism of metoprolol.

This study addresses a critical gap by developing and validating a simple, multi-parameter PRS model for risk stratification in children with VVS who receive metoprolol. By integrating several readily obtainable variables, the PRS model overcomes the limited discriminatory power of single-parameter prognostic models while remaining straightforward enough for routine clinical use. Kaplan–Meier analysis with log-rank testing demonstrated significantly higher cumulative syncopal recurrence rates in the high-risk PRS stratum than those in the low-risk stratum (*p* < 0.01), confirming the model’s utility for risk stratification. These findings might suggest a possible tailoring therapy that high-risk patients with VVS might benefit from an intensified intervention, such as extended metoprolol courses, combination pharmacotherapy, and closer follow-up, to improve long-term prognosis. However, this tailoring therapy is only hypothesis-generating and merits further validation through future prospective studies.

Additionally, all variables incorporated into the model were extracted from continuous Holter recording. This technique is both well established in routine practice and non-invasive, offering high signal stability and excellent pediatric tolerability, thereby enhancing acceptance among children and their families. The time-domain HRV indices (SDNN, TR) and the frequency-domain component (VLF) reliably reflect autonomic balance. In our study, each of these three metrics emerged as an independent predictor of long-term outcome in children with VVS treated with metoprolol.

However, this study has several limitations. It is retrospective, conducted in a single center, and has a small sample size. Secondly, no control group was established in this study, making it impossible to clarify the relative efficacy and incremental value of the metoprolol for VVS through direct comparison. Consequently, it is difficult to rule out the interference of factors such as the natural course of the disease on the outcomes. Furthermore, retrospective studies are inherently prone to significant selection bias, particularly indication bias—the baseline clinical characteristics and treatment decisions of enrolled patients may be strictly restricted by clinical indications. This results in a study population that cannot adequately represent patients in broader clinical settings, thereby limiting the generalizability of the findings. Meanwhile, the study failed to fully identify and control potential confounding factors (such as baseline comorbidities, previous treatment history, lifestyle factors, and physiological factors). In addition, there is a relatively high rate of loss to follow-up. Due to the limited sample size, external validation of the model’s efficiency and PRS-based risk stratification was not possible. As a result, the generalizability of the model in different populations and medical settings cannot be evaluated, and its clinical utility awaits further verification. To address these limitations, future research should validate these findings through multicenter, large-sample, prospective studies. These studies should include external validation cohorts and the collection of long-term follow-up data. This will provide a more reliable basis for clinical guidance.

## 5. Conclusions

This study demonstrates that the HRV indices—specifically the time-domain measures SDNN and TR, as well as the frequency-domain measure VLF—are influential factors related to the prognosis of VVS patients treated with metoprolol. A PRS model constructed based on these HRV indicators can be used for risk stratification, referring to long-term prognosis. Patients with lower baseline SDNN, higher TR, and higher VLF were found to have a lower long-term cumulative syncope recurrence rate and a better prognosis after metoprolol treatment. These findings highlight the key role of the HRV-based PRS model in assessing the prognosis of metoprolol-treated VVS patients.

## Figures and Tables

**Table 1:** Baseline characteristics of children with vasovagal syncope.

Variables	Baseline Value	
number (n)	97	
sex (female/male, n)	49/48	
age (year)	12.0 (11.0, 14.0)	
diastolic blood pressure (mmHg)	67.0 (60.0, 75.5)	
systolic blood pressure (mmHg)	111.0 (106.0, 121.0)	
heart rate (bpm)	81.0 ± 9.0	
SDNN (ms)	139.9 ± 33.5	
SDANN (ms)	131.0 (102.5, 162.5)	
ASDNN (ms)	69.1 ± 19.4	
rMSSD (ms)	43.0 (32.5, 60.5)	
pNN50 (%)	17.0 (9.8, 26.8)	
TR	29.0 (27.0, 31.0)	
HF (ms)	23.0 (17.3, 32.7)	
LF (ms)	29.0 (23.4, 34.6)	
VLF (ms)	43.1 (35.8, 50.8)	
LF/HF (ms)	1.34 (1.1, 1.6)	
hemodynamic types of VVS (n)	vasopressor	84
	cardioinhibitory	7
	mixed	6
syncope triggers (n)	postural changes	28
	standing for a long time	26
	stifling environment	7
	emotional condition	5
	exercise (walking/running or jumping/stairs/higher leg lifts)	10 (5/2/2/1)
	other situations (turning/blood draws/intramuscular injections/combing hair)	4 (1/1/1/1)
	no triggers	17
precursor symptoms of syncope (n)	blackouts and blurred vision	38
	dizziness, tinnitus	43
	pale and weak	25
	without precursor symptoms	8
total number of syncopal episodes before treatment	4 (2, 6)	

ASDNN, Average standard deviation of NN intervals for each 5 min segment; bpm, beat per minute; HF, high frequency; LF, low frequency; LF/HF, low frequency/high frequency; mmHg, millimeters of mercury; ms, millisecond; pNN50, percentage difference between adjacent NN intervals > 50 ms; rMSSD, root mean square of the successive NN interval differences; SDANN, standard deviation of the averages of NN intervals in all 5 min segments of the entire recording; SDNN, standard deviation of NN intervals; TR, triangular index; VLF, very low frequency.

**Table 2:** Univariate Cox proportional risk regression for long-term prognosis of vasovagal syncope in children treated with metoprolol.

Variables	HR (95% CI)	*p*
number [n (%)]	-	-
diastolic blood pressure (mmHg)	0.99 (0.96–1.02)	0.65
systolic blood pressure (mmHg)	1.00 (0.98–1.02)	0.93
heart rate (bpm)	0.96 (0.92–0.99)	0.02
age (years)	1.00 (0.98–1.00)	0.96
HF (ms)	1.00 (0.98–1.01)	0.65
SDNN (ms)	1.00 (1.01–1.03)	0.01
rMSSD (ms)	1.01 (0.99–1.02)	0.01
SDANN (ms)	1.01 (0.99–1.01)	0.03
VLF (ms)	0.99 (0.98–1.00)	0.17
LF (ms)	0.99 (0.97–1.00)	0.54
LF/HF	0.61 (0.32–1.10)	0.12
pNN50 (%)	1.00 (0.99–1.05)	0.01
TR	1.00 (0.99–1.10)	0.01

ASDNN, Average standard deviation of NN intervals for each 5 min segment; CI, confidence interval; HF, high frequency; LF, low frequency; LF/HF, low frequency/high frequency; HR, Hazard Ratio; mmHg, millimeters of mercury; ms, millisecond; pNN50, percentage difference between adjacent NN intervals > 50 ms; rMSSD, root mean square of the successive NN interval differences; SDANN, standard deviation of the averages of NN intervals in all 5 min segments of the entire recording; SDNN, standard deviation of NN intervals; TR, triangular index; VLF, very low frequency.

**Table 3:** Multivariate Cox proportional risk regression for long-term prognosis of vasovagal syncope in children treated with metoprolol.

Variables	HR (95% CI)	*p*	β
SDNN (ms)	1.03 (1.01–1.05)	0.001	0.03
VLF (ms)	0.98 (0.96–0.99)	0.049	−0.02
TR	0.90 (0.84–0.97)	0.003	−0.10
ASDNN (ms)	1.01 (0.99–1.04)	0.297	0.01

PRS = 0.03 × SDNN − 0.02 × VLF − 0.1 × TR; ASDNN, Average standard deviation of NN intervals for each 5 min segment; HR, hazard ratio; ms, millisecond; PRS, prognostic risk score; SDNN, standard deviation of NN intervals; VLF, very low frequency; TR, triangular index; β, regression coefficients.

## Data Availability

The original contributions presented in this study are included in the article. Further inquiries can be directed to the corresponding authors.

## Abbreviations

The following abbreviations are used in this manuscript:

ASDNN	mean of the standard deviation of NN intervals for each 5 min segment
AUC	Area under curve
Bca	Bias-corrected and accelerated
BMI	Body mass index
CI	Confidence interval
CS	Cardiogenic syncope
DCA	Decision curve analysis
ECG	Electrocardiogram
HF	High frequency
HR	Hazard ratios
HRV	Heart rate variability
HUTT	Head-up tilt test
IQR	Interquartile range
LASSO	Least absolute shrinkage and selection operator
LF	Low frequency
MPV	Mean platelet volume
NMS	Neurally mediated syncope
pNN50	Adjacent NN intervals > 50 ms
PRS	Prognostic Risk Score
QTcd	Corrected QT dispersion
rMSSD	Root mean square of the successful NN interval differences
ROC	Receiver operating characteristics
SDANN	Standard deviation of the averages of NN intervals in all 5 min segments of the entire recording
SDNN	Standard deviation of all NN intervals
TR	Triangular index
VLF	Very low frequency
VVS	Vasovagal syncope
